# Charcot Marie Tooth disease pathology is associated with mitochondrial dysfunction and lower glutathione production

**DOI:** 10.1007/s00018-025-05612-0

**Published:** 2025-02-07

**Authors:** Nafisa R. Komilova, Plamena R. Angelova, Elisa Cali, Annarita Scardamaglia, Ulugbek Z. Mirkhodjaev, Henry Houlden, Noemi Esteras, Andrey Y. Abramov

**Affiliations:** 1 UCL Queen Square Institute of Neurology, Queen Square, London, WC1N 3BG UK; 2 Department of Biophysics, National University of Uzbekistan, Tashkent, Uzbekistan; 3 Center for High Technologies, Tashkent, Uzbekistan; 4 Neurochemistry Research Institute, Department of Biochemistry and Molecular Biology, School of Medicine, Complutense University of Madrid, Madrid, Spain; 5 CIBERNED, Network Center for Biomedical Research in Neurodegenerative Diseases, Madrid, Spain

**Keywords:** CMT (Charcot Marie Tooth disease), Mitochondria, ATP, Reactive oxygen species, Glutathione

## Abstract

Charcot Marie Tooth (CMT) or hereditary motor and sensory neuropathy is a heterogeneous neurological disorder leading to nerve damage and muscle weakness. Although multiple mutations associated with CMT were identified, the cellular and molecular mechanisms of this pathology are still unclear, although most of the subtype of this disease involve mitochondrial dysfunction and oxidative stress in the mechanism of pathology. Using patients’ fibroblasts of autosomal recessive, predominantly demyelinating form of CMT—CMT4B3 subtype, we studied the effect of these mutations on mitochondrial metabolism and redox balance. We have found that CMT4B3-associated mutations decrease mitochondrial membrane potential and mitochondrial NADH redox index suggesting an increase rate of mitochondrial respiration in these cells. However, mitochondrial dysfunction had no profound effect on the overall levels of ATP and on the energy capacity of these cells. Although the rate of reactive oxygen species production in mitochondria and cytosol in fibroblasts with CMT4B3 pathology was not significantly higher than in control, the level of GSH was significantly lower. Lower level of glutathione was most likely induced by the lower level of NADPH production, which was used for a GSH cycling, however, expression levels and activity of the major NADPH producing enzyme Glucose-6-Phosphate Dehydrogenase (G6PDH) was not altered. Low level of GSH renders the fibroblast with CMT4B3 pathology more sensitive to oxidative stress and further treatment of cells with hydroperoxide increases CMT patients’ fibroblast death rates compared to control. Thus, CMT4B3 pathology makes cells vulnerable to oxidative stress due to the lack of major endogenous antioxidant GSH.

## Introduction

Charcot Marie Tooth (CMT) is the most common inherited neurological disorder known as a hereditary motor and sensory neuropathy affecting approximately 2.6 million people worldwide [1]. CMT is a heterogeneous genetic disorder, meaning that mutations in different genes may result in similar clinical symptoms [2].

CMT has multiple patterns of inheritance such as X-linked, autosomal recessive and autosomal dominant [3]. More than 30 genes have been identified as targets of mutations that cause CMT neuropathy [4]. CMT4B3 subtype is an ultra-rare form of CMT characterized as an autosomal recessive, predominantly demyelinating form accounting for less than 10% of all CMT cases, with less than a hundred currently reported cases [5]. CMT4B3 is associated with the loss of function biallelic mutations in the myotubularin-related 5 (MTMR5), also called SET binding factor 1 (SBF1) gene [6, 7]. The protein MTMR5/SBF1 is a pseudo phosphatase which is thought to regulate endo-lysosomal trafficking in line with other MTMRs [6].

Mitochondrial dysfunction is associated with most of the neurological conditions including CMT [8–10]. Changes in the activity of mitochondrial electron transport chain complexes was also shown for CMT4B3 [6]. However, it is not clear if these changes could have an effect on mitochondrial energy metabolism. Further, mitochondrial function has also been linked to redox biology and oxidative stress in pathology [11].

Reactive oxygen species overproduction and oxidative stress are shown to be one of the major triggers for development of neurological conditions [12, 13]. For some of the inherited forms of CMT an increased level of ROS production has also been shown [14] that induces oxidative stress resulting in the decrease of major endogenous antioxidant glutathione [15]. However, it was mostly shown for the CMT-linked mutation (GDAP1) which encodes a protein associated with mitochondrial function.

In order to identify the molecular and cellular mechanism of CMT4B3 pathology we studied the effects of CMT4B3 mutations on mitochondrial energy metabolism and redox balance in patients’ fibroblasts. We have found that mitochondrial membrane potential in most of the CMT4B3 patients’ fibroblasts was lower than in control cells and pathology in mitochondrial electron transport chain was partially compensated by pumping protons by the F_0_-F_1_-ATPase. However, it all leads to only a minor decrease in ATP level in patients’ cells. All cells with CMT4B3 pathology had a significantly lower level of GSH although the rate of reactive oxygen species production was similar and, in some cells, even lower that in control.

## Materials and methods

### Cell lines

Experiments were performed with 6 different cell lines. The two control lines #477, #754, and four patient cell lines #946, #1113, #1132 and #1159 was obtained from UK Cell Bank. Fibroblasts were generated from a 4-mm skin punch biopsy taken under local anaesthetic following local ethical approval. All patients gave full informed consent. We have ethical approval for investigating patients with informed consent and taking skin samples for research approved by University College London Hospital ethics committee (Number: 07/N018). Biopsies were dissected into ~ 1-mm pieces and cultured in DMEM, 10% FBS, and 1% GlutaMAX until fibroblasts were seen to grow out from the explants. When fibroblasts reached confluency, they were detached from culture dishes using TrypLE Express (Invitrogen) and transferred to larger culture vessels for further expansion and cryopreservation. Age, sex, clinical diagnosis, and gene mutations of the donors are depicted in Table 1.

**Table 1:** Donors' information

Line	Diagnosis	Gene	Mutation	Sex	Age at sampling	Age at onset
#477 Control	Healthy Control			Male	31	
#754 Control	Healthy Control			Female	30	
#946	CMT 4B3	SBF1/CMT4B3	c.3493_3494dupTA p.P1166TfsX5 paternal: c.5474_5475delTG p.V1825GfsX27	Male		
#1113	CMT—complex	SBF1/CMT4B3	c.5477_5478del	Male	34	20
#1132	CMT 4B3	SBF1/CMT4B3	c.2828C > T (p.Thr943Met) + c.1432G > A (p.Glu478Lys)	Female	30	17
#1159	CMT4B3	SBF1/CMT4B3	c.305_306delCA	Female	24	

###  Live imaging

All experiments were done in commercial HBSS 1X (with calcium and magnesium) (ThermoFisher) supplemented with 10 mM HEPES and adjusted to pH 7.4.

### Measurement of mitochondrial membrane potential and mitochondrial mass

Cells were incubated at room temperature for 40 min with 25 nM tetramethylrhodamine methyl ester (TMRM), and when necessary for mitochondrial mass measurements with Calcein-AM (1 μM, Molecular Probes, ThermoFisher) and 0.005% Pluronic. Images were acquired using a Zeiss 710 VIS CLMS confocal microscope equipped with a META detection system and an × 40 oil immersion objective (Zeiss, Oberkochen, Germany). The 560 nm laser line was used to excite the TMRM and the 488 nm to excite calcein. Emitted fluorescence was measured above 580 nm for TMRM and between 500 and 540 nm for calcein, keeping the power laser at minimum to avoid phototoxicity. Z- stacks were acquired for the calculations of the mitochondrial membrane potential and mass. Images were analysed using Volocity 3D Image Analysis Software (PerkinElmer, Waltham, MA, USA). Data was obtained from n = 9–38 Z-Stacks in 6–12 independent experiments done with different inductions and was normalized to control cells in all of them. For ΔΨm maintenance experiments, a single focal plane was selected and TMRM intensity was measured during the appropriate amount of time. TMRM was used in the redistribution mode, so a reduction in TMRM signal represents mitochondrial depolarization. Zeiss software was used for the analysis of the experiments. Basal TMRM levels were taken as 100% and remaining TMRM fluorescence in the mitochondria after complete depolarization caused by FCCP was taken as 0%. Data was obtained from n = 40–60 cells analysed in 3–5 independent experiments done with different inductions.

### Measurement of NADH redox state

NADH autofluorescence was measured using an epifluorescence-inverted microscope equipped with a × 40 oil objective. A Xenon arc lamp passed through a monochromator was used to provide excitation light at 360 nm. Emitted light was reflected through a 455 nm long-pass filter to a cooled CCD camera. Images were acquired and analysed using Andor Software. After recording basal autofluorescence, 1 μM FCCP was added to completely depolarize the mitochondria and oxidize the mitochondrial pool of NADH to NAD^+^, which is no longer fluorescent. This point was taken as 0%. 1 mM NaCN was then added to inhibit respiration and allow the regeneration of the mitochondrial pool of NADH (100%). NADH pool was calculated as the difference between maximum and minimum values of autofluorescence in the mitochondria. NADH redox index was calculated as the % represented by the basal levels when extrapolating its value in the 0–100% range generated by FCCP and NaCN respectively. A total number of n = 100–200 cells were analysed in 4–7 independent experiments (N).

### Measurement of ATP

For the monitoring of ATP levels, cells were transfected with the mitochondrial-targeted ATP indicator AT1.03, designed by [16]. This genetically encoded FRET indicator allows the visualization of the dynamics of ATP in real time. Briefly, this probe contains a variant of the cyan fluorescent protein (CFP) and a variant of yellow fluorescence protein (YFP). When ATP is not bound to the probe, both fluorescent proteins are separated and the FRET efficiency is low, so fluorescence emitted from the CFP is the mainly detected. In contrast, when ATP binds to the probe, the two fluorescence proteins come close to each other, increasing FRET efficiency, and as a result, YFP signal. Thus, the YFP/CFP emission ratio is used to evaluate ratiometrically the levels of ATP. Measurement of ATP levels with mitoAT1.03 probe was performed on the confocal microscope Zeiss 710 LSM with an integrated META-detection system using a 40 × oil-immersion objective. CFP was excited with 405 nm laser, and its emission was detected between 460–510 nm. Emission from YFP was detected between 540–600 nm. Cells were transfected using the Effectene transfection reagent from Qiagen (Hilden, Germany), following the manufacturer's indications. Data was obtained from n = 13–37 cells in 4–7 independent experiments (N) and was normalized to control cells.

### MagFura-2 measurements

To assess the ATP levels, which correlate with Mg^2+^ changes, [Mg^2+^] was imaged using MagFura-2 AM. Fluorescence images were acquired (10 s interval) on an epifluorescence inverted microscope equipped with a 20 × fluorite objective (excitation at 340 and 380 nm). The emitted light was reflected through a 515 nm long-pass filter to a cooled CCD camera (Retiga; QImaging) and digitised to 12-bit resolution (Cairn Research, UK). Andor iQ3 was employed for data collection and analysis.

### ROS production

Cytosolic ROS production was monitored in single cells using the superoxide indicator dihydroethidium (DHE, 2 μM, Molecular Probes, Thermo Fisher Scientific), which shows blue fluorescence in the cytosol until oxidized, when it intercalates within the DNA, staining the nucleus fluorescent red. Live-imaging experiments were performed in an epifluorescence inverted microscope using a Xenon arc lamp passed through a monochromator to provide excitation light at 530 nm. Emitted light was reflected through a 605 nm long-pass filter to a cooled CCD camera. Cells were loaded with DHE and basal rate of ROS production, measured as the rate of increase in red DHE fluorescence, was immediately recorded for several minutes. Images were captured and analysed using IQ3 software from Andor. Around n = 100–200 cells were analysed in at least four independent experiments (N = 4). Mitochondrial ROS production was analysed using the mitochondrially-targeted dye MitoTracker Red CM-H_2_XRos (Molecular Probes, Thermo Fisher Scientific). Cells were loaded for 20 min with the dye, and measurements were done on the confocal microscope Zeiss 710 LSM with an integrated META-detection system using a 40 × oil-immersion objective. The dye was excited with 561 nm laser, and emission was detected above 580 nm and recorded for several minutes. The rate of increase in red fluorescence for each whole field was analysed using Volocity 3D Image Analysis Software (PerkinElmer, Waltham, MA, USA). 4–7 fields containing at least n = 100 cells each were analysed in 4–6 independent experiments (N).

### GSH level assessment

Cells were incubated with 50 μM monochlorobimane (MCB) (Molecular Probes, Invitrogen) for 40 min in HEPES buffered salt solution prior to imaging. Cells were then washed with HEPES buffered salt solution and images of the fluorescence of the MCB-GSH were acquired using a Zeiss UV–vis 710 CLSM with excitation at 405 nm and emission at 435–485 nm.

### Western blots

Protein extracts were collected in ice-cold RIPA lysis buffer supplemented with protease and phosphatase inhibitors (Thermo Fisher Scientific, Paisley, UK). Samples were snap-frozen, sonicated, and centrifuged at 22 000 g, and protein content of the extracts was determined by the Pierce™ BCA protein assay (Thermo Fisher Scientific, Paisley, UK). 15–20 μg of protein was then fractionated on an SDS polyacrylamide gel (4%–12%) (Thermo Fisher Scientific, Paisley, UK), transferred to an Immobilon-P PVDF membrane (Merck), and blocked with 5% non-fat milk. Membranes were incubated overnight with the corresponding primary antibodies diluted in 5% bovine albumin serum: Glutathione synthetase (GSS) (mouse) 1: 1000, Glutathione reductase (mouse) 1: 1000, Glucose 6-Phosphate dehydrogenase (G6PDH) (rabbit) 1: 1000 and anti-actin (rabbit) 1: 5000, all from Cell Signaling Technologies (Danvers, MA, USA) and afterward with the corresponding specie-specific HRP-conjugated secondary antibodies. The luminol-based Pierce™ ECL Western Blotting Substrate (Thermo Fisher Scientific) was used to detect the HRP activity. Protein band densities were quantified using ImageJ (NIH, Bethesda, MD, USA) after scanning the X-ray films. In order to compare the results between experiments, in all cases results were normalized to t = 0.

#### G6PDH activity

Glucose 6 Phosphate Dehydrogenase Activity was estimated using a fluorometric kit by Abcam (ab176722) following manufacturer’s instructions. Beforehand, fibroblasts were harvested and homogeneized in PBS, and protein content was quantified with the BCA Protein Assay Kit (Thermo Fisher). A similar amount of protein was loaded per cell line. 8 samples per line were analysed in 4 independent experiments.

#### Cell death

Cells were seeded in 6-well plates at a density of 1 × 10^4^ cells/well and treated with t-BHP (50 μM for 24 h). For cell death measurements after treatment with t-BHP, cells were washed twice with HBBS and loaded with 20 μM propidium iodide and 10 μM Hoechst 33,342. While Hoechst is a fluorescent dye that stains chromatin DNA (blue, total number of cells), propidium iodide is only permeable to dead cells and shows red fluorescence, so it is possible to calculate the percentage of dead cells (ration blue to red). Fluorescence measurements were obtained on an epifluorescence-inverted microscope equipped with a 20 × fluorite objective. Excitation light [for Hoechst 33,342 380 nm and for PI (530 nm)] provided by a Xenon arc lamp. Emitted fluorescence light was reflected through a 515-nm long-pass filter to a cooled CCD camera (Retiga, QImaging, Canada). A total number of 800–1000 cells were counted in 4–5 different fields per coverslip. Experiments were repeated 3 times with separate independent cultures (N = 3).

#### Statistics

All histograms represent the average ± SEM. Statistical analysis (One-way ANOVA followed by Tukey post-hoc correction) was performed in Origin Pro 2016 software. Differences were considered to be significant if p < 0.05.

## Results

### CMT4B3 pathology change mitochondrial membrane potential in patient’s fibroblasts

**Fig. 1:**
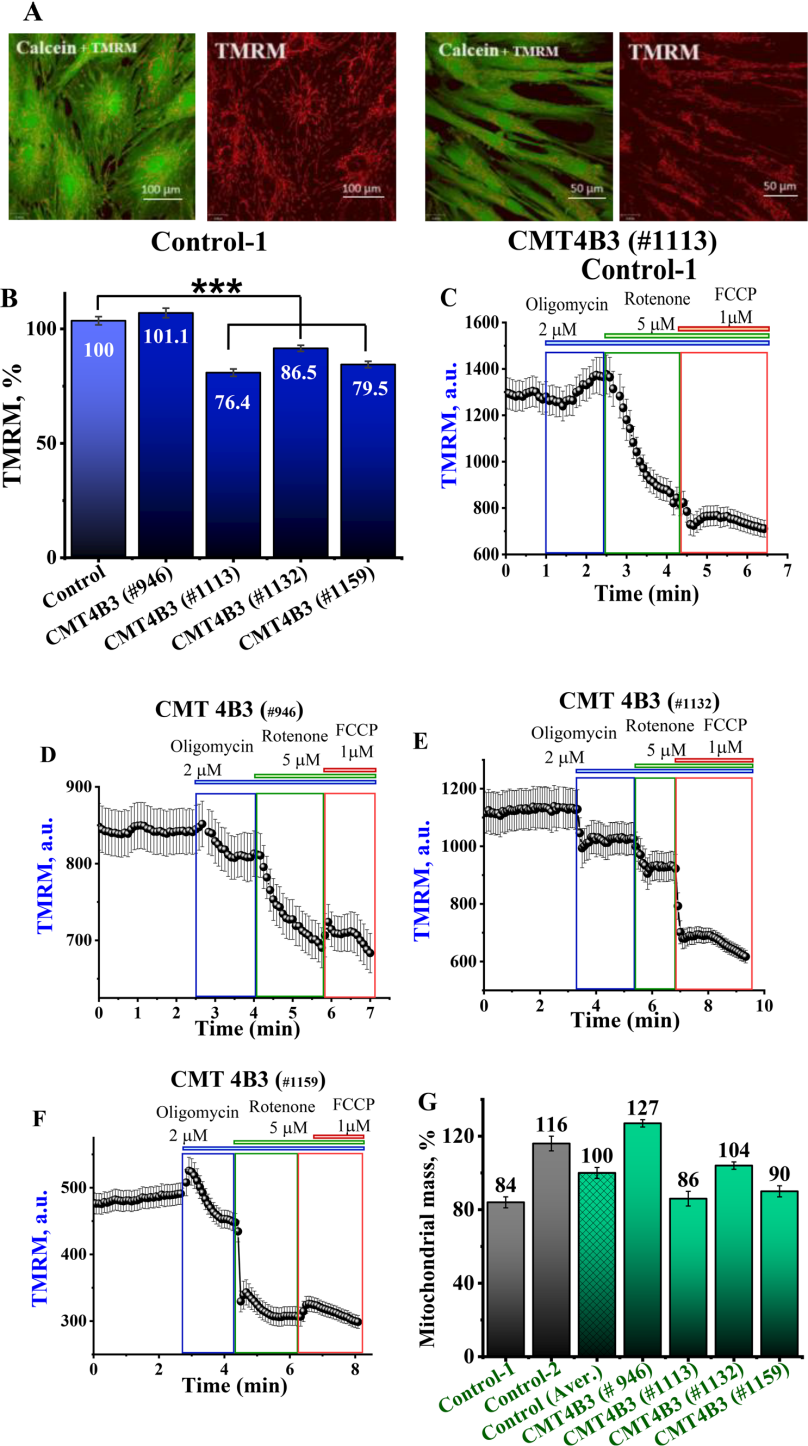
Mitochondrial membrane potential is lower in patient’ cells with CMT4B3 mutation. **A**. Representative images from live imaging experiments showing the TMRM (red) and calcein (green) staining. Scale bars 50 μM, 100 μM. **B** Fibroblasts from patients with CMT4B3 mutation showed a significant decrease in ΔΨm compared to controls (***p < 0.001). **C**–**F** Time-course recordings of TMRM fluorescence changes, corresponding to the maintenance of ΔΨm in the presence of inhibitors of different ETC complexes. Oligomycin (inhibitor of complex V), rotenone (inhibitor of complex I) and FCCP ( mitochondrial uncoupler) to the maintenance of ΔΨm in patients’ and control cell lines. **G** Mitochondrial mass estimated by the percentage of cell volume occupied by mitochondria (stained with TMRM) in relation to the total volume of the cell (stained with calcein), (n = 40–60; N = 3–5). Data are represented as mean ± SEM

Mitochondrial membrane potential is an indicator of functional state of mitochondria. Using TMRM (20 nM) as a fluorescent probe for detection of mitochondrial membrane potential we have found that CMT4B3 pathology induce 15–20% decrease in Δψm (Fig. 1A, B). Any pathology in the electron transport chain can be compensated by other mitochondrial mechanisms to maintain Δψm. To identify the mechanism of maintenance of mitochondrial membrane potential in control and CMT4B3 patients’ fibroblasts we used mitochondrial inhibitors and uncoupler. Thus, application of inhibitor of complex V oligomycin at 2 μg/ml induced a small increase in the TMRM signal in control fibroblasts while in CMT4B3 pathology it resulted in mitochondrial depolarisation suggesting that in CMT4B3 pathology F_0_-F_1_-ATPase was working in a reverse mode, as ATPase, pumping protons for Δψm maintenance (Fig. 1C–F). Importantly, this reverse activity of complex V can completely compensate Δψm to control values in some cell lines (Fig. 1B, D). Subsequent application of the inhibitor of mitochondrial complex I rotenone (5 μM) to these cells induced a decrease in TMRM fluorescence in both control and patient’s fibroblasts (Fig. 1C–F) that suggests that most of the mitochondrial membrane potential is maintained by complex I and that the function of this complex in CMT4B3 pathology is lower than in control. Application of the mitochondrial uncoupler FCCP (1 μM) at the end of the experiment induced a complete depolarisation of mitochondria and the difference in the responses to the Δψm uncoupler in control and CMT4B3 fibroblasts show impact of mechanisms other than complex I and complex V in the maintenance of Δψm (Fig. 1C–F). Thus, CMT4B3 pathology leads to mitochondrial depolarisation and a change in the mechanism of maintenance of Δψm.

Mitochondrial dysfunction could be compensated in the probands’ cells by the increase of the mitochondrial number. To identify mitochondrial mass, we have used the percentage of colocalization of mitochondrial signal (TMRM) and the total volume of fluorescence from each cell (Calcein). We have not found any direct corelation of the CMT pathology and colocalization coefficients. Moreover, changes in mitochondrial mass varied broadly depending on patient’s cell line (Fig. 1G).

### CMT4B3 pathology led to changes in mitochondrial NADH redox level

**Fig. 2:**
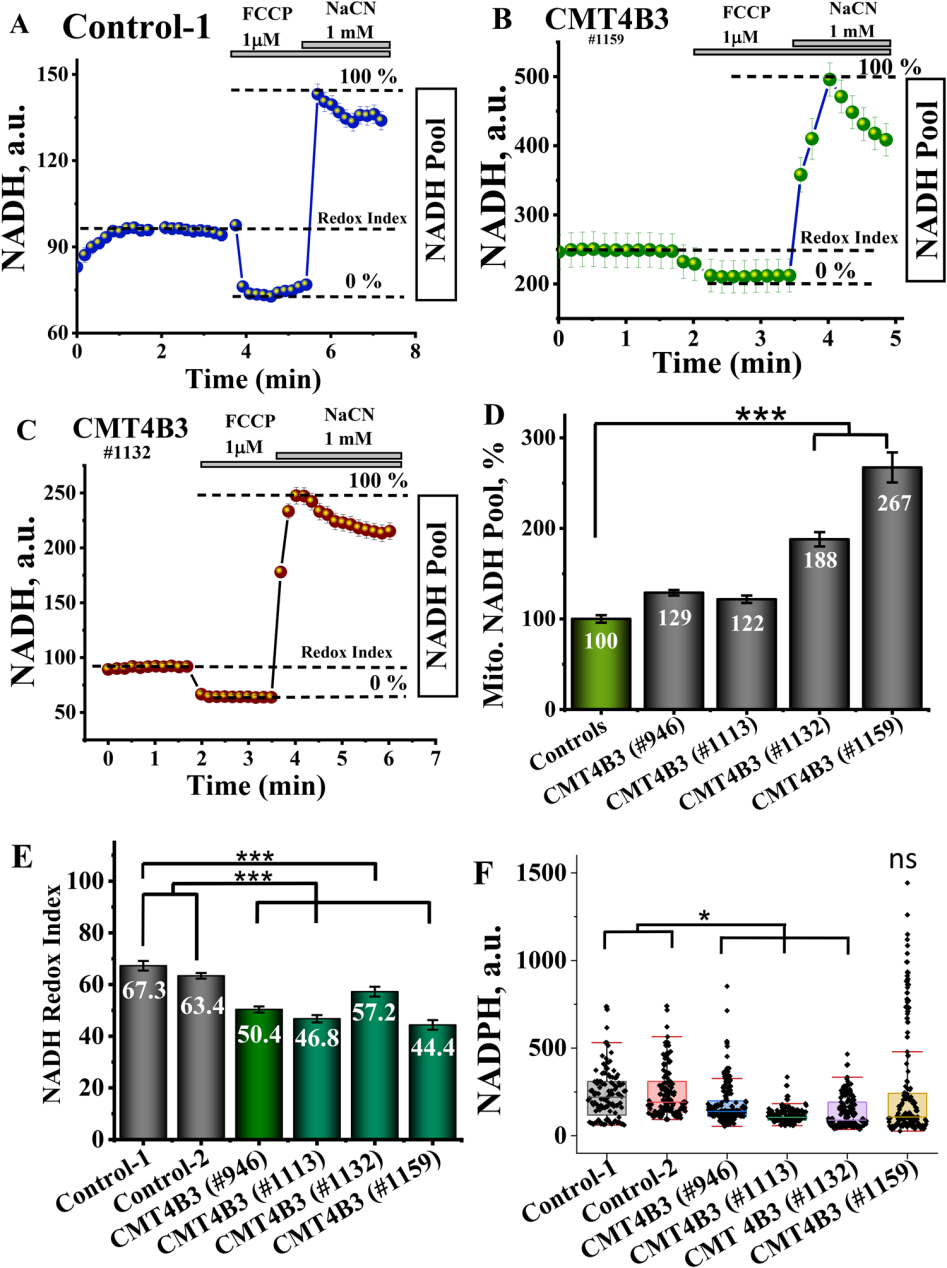
NADPH and NADH Redox indexes are lower in fibroblasts from patients’ with CMT4B3 mutation. **A**–**C** Representative traces of NADH status experiments. Basal autofluorescence of NADH was measured prior to the addition of FCCP to completely depolarize mitochondria, stimulating mitochondrial respiration and causing the complete oxidation of NADH. This point was considered 0%. NaCN was then added to inhibit respiration and allow for complete regeneration of mitochondrial NADH pool, taken as 100%. Basal NADH redox state (%) was extrapolated from the 0–100% range and mitochondrial NADH pool was calculated as the difference between min and max autofluorescence signals. **D** The mitochondrial NADH pool in fibroblasts of patients with CMT4B3 pathology exhibited similarities to the control group, and in two cell lines, it was even higher than that observed in the control (***p < 0.001). **E** NADH Redox Index is lower in patients carrying the CMT4B3 mutation (***p < 0.001), suggesting the activated respiration. **F** The level of NADPH in the patients' cells was notably reduced (*p < 0.05). n = 100–200; N = 4–7 independent experiments. Data are represented as mean ± SEM

Mitochondrial redox activity in living cells can be assessed by the measurements of NADH autofluorescence. Maximal activation of mitochondrial respiration by the uncoupler FCCP (1 μM) leads to the minimal level of NADH in mitochondria (taken as 0) while inhibition of the mitochondrial respiration with 1 mM NaCN blocks the consumption of NADH and leads to the maximal rise of NADH autofluorescence (taken as 100; Fig. 2A–C). These applications allow us to assess the relative NADH level in mitochondria, but also the NADH redox index (the balance between production and consumption of NADH in mitochondria—Fig. 2A–C) [17]. Importantly, the residual NADH autofluorescence after the application of FCCP can also be taken to assess NADPH levels [18]. In our experiments mitochondrial NADH pool in fibroblasts of the patients with CMT4B3 pathology was similar to control and in two cell lines was even higher than in control (Fig. 2D), suggesting that limitation in NADH is not the reason for mitochondrial dysfunction in this pathology. However, CMT4B3 pathology led to a decrease in the NADH redox index (Fig. 2E) in all patients’ fibroblasts that may be an indicator of higher activity of mitochondrial respiration in these cells. In contrast to the NADH pool, the level of NADPH in the patients’ cells was significantly lower (Fig. 2F).

Thus, shift of the NADH redox index down with higher NADH pool in CMT4B3 fibroblasts suggest higher consumption of NADH–activation of mitochondrial respiration that in combination with lower Δψm suggest mitochondrial uncoupling.

### CMT4B3 pathology has a minor implication to the ATP contents in the cells

**Fig. 3:**
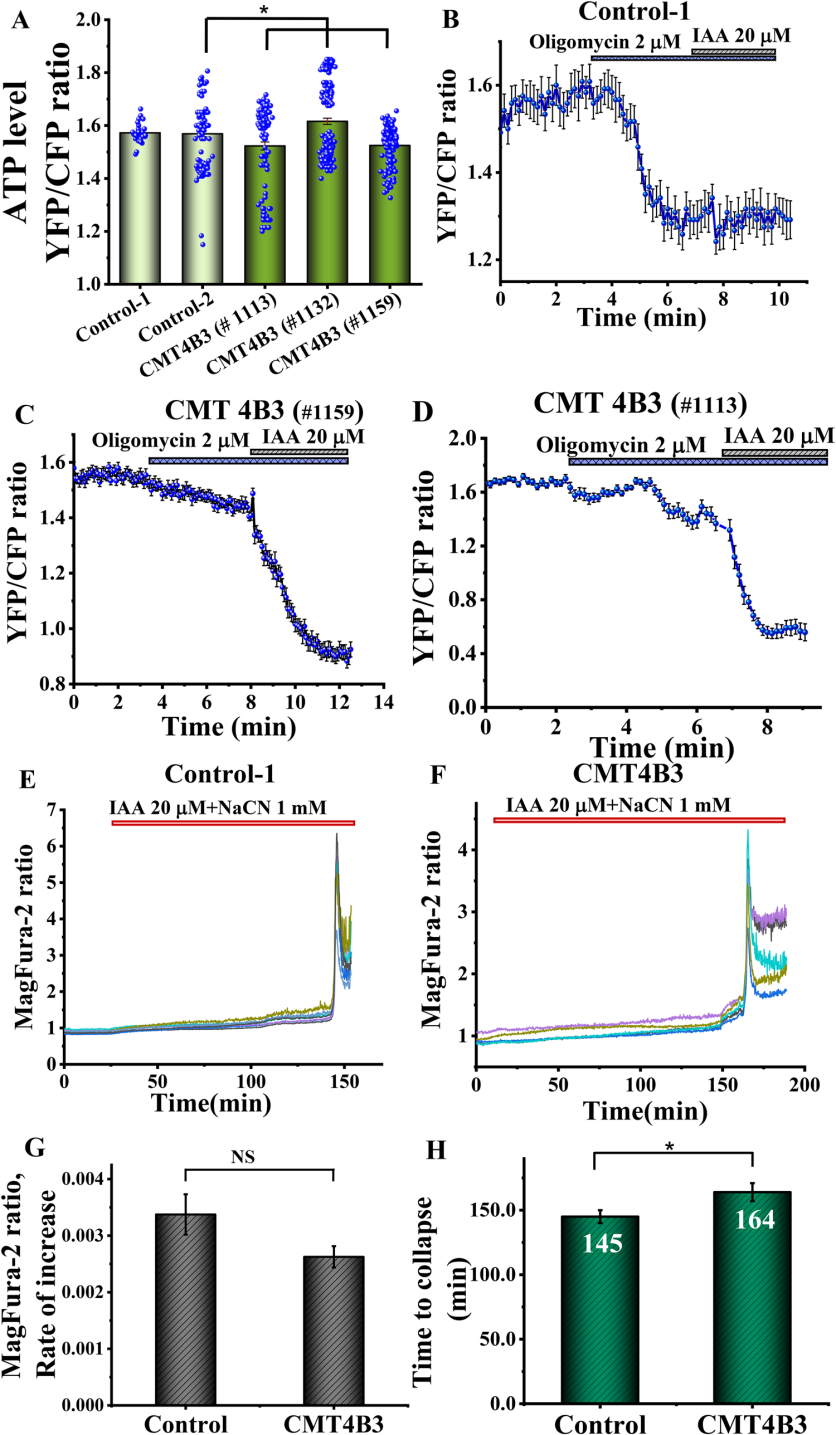
ATP levels are reduced in patient’ cells, but this does not result in energy collapse in the cells of the patients with CMT4B3 mutation. **A** Basal measurements of ATP levels show minimal difference between controls and patient cells (*p < 0.05). **B**–**D** Representative traces showing ATP live measurements. Cells were transfected with AT1.03 probe allowing the visualization of mitochondrial ATP in vivo. After measurement of basal levels of ATP (ratio YCF/CFP), oligomycin was added to inhibit complex V. The subsequent reduction in ATP levels can be attributed to ATP generated by oxidative phosphorylation (OXPHOS). Decrease in ATP levels caused by the following addition of iodoacetic acid can be attributed to ATP generated by glycolysis. 3E, F. Representative Mag-Fura-2 traces of control (**E**) and patient cells (**F**) after treatment with Iodoacetic acid (IAA) and sodium cyanide (NaCN), to block oxidative phosphorylation and glycolysis. 3 G, H. Quantification of the rate of ATP consumption and the time to collapse in cells exposed to IAA and NaCN. n = 13–37; N = 4–7 experiments; Data are represented as mean ± SEM

The relative levels of ATP in control and patient’s fibroblasts were assessed using the genetically encoded ATP probe AT1.03 [16]. Despite the significant changes in Δψm and NADH redox index, CMT4B3 pathology had bidirectional effect on the basal level of ATP in the patient’s cells and increase it in one line and decrease in two other lines of fibroblasts (Fig. 3A). ATP is produced in the cells mainly by glycolysis and oxidative phosphorylation. To estimate the impact of these processes on the total ATP production in control and patient’s cells we added inhibitor of oxidative phosphorylation −2 μg/ml oligomycin, which induced profound decrease in ATP level in control cells (n = 20–30 cells; Fig. 3B). Subsequent addition of inhibitor of glycolysis iodoacetic acid (IAA, 20 μM) induced only a minor (3–5%) decrease in ATP level suggesting that under these conditions most of the ATP in control fibroblasts is produced in oxidative phosphorylation. However, in the majority of the cells with CMT4B3 pathology the effect of oligomycin on the ATP level was much smaller than in control but IAA induced much more profound effect, suggesting that in patient’s fibroblasts ATP is produced mainly in glycolysis (Fig. 3C, D).

When ATP is hydrolysed, Mg^2+^ is released from the Mg-ATP complex, and the changes in the cellular Mg^2+^ using sensitive fluorescent indicator MagFura-2, can be used for indirect assessment for the consumption rate of ATP [19]. MagFura-2 is also a low-affinity Ca^2+^ indicator that can help detecting high cytosolic calcium rise in the time of cellular lysis, i.e. the energetic collapse due to a total cellular ATP depletion and the inability of the cell to maintain Ca^2+^ homeostasis, enables the estimation of the cellular energy capacity. In our experiments inhibition of the cellular ATP production with inhibitor of mitochondrial respiration 1 mM NaCN (that also inhibit coupled to respiration oxidative phosphorylation) and 20 μM IAA (glycolysis) induced slow and progressive increase of the MagFura-2 ratio indicating the rate of ATP consumption in these fibroblasts (Fig. 3E–G). Energy collapse and massive increase in MagFura-2 ratio in control and patient’s fibroblasts was observed after ~ 150 min after application of inhibitors with increased time to collapse in fibroblasts with CMT4B3 pathology (Fig. 3E–H). Thus, despite the mitochondrial dysfunction and changes in ATP levels in patient’s cells it does not lead to energy collapse.

### Production of reactive oxygen species is not increased in mitochondria and cytosol of fibroblasts with CMT4B3 pathology

**Fig. 4:**
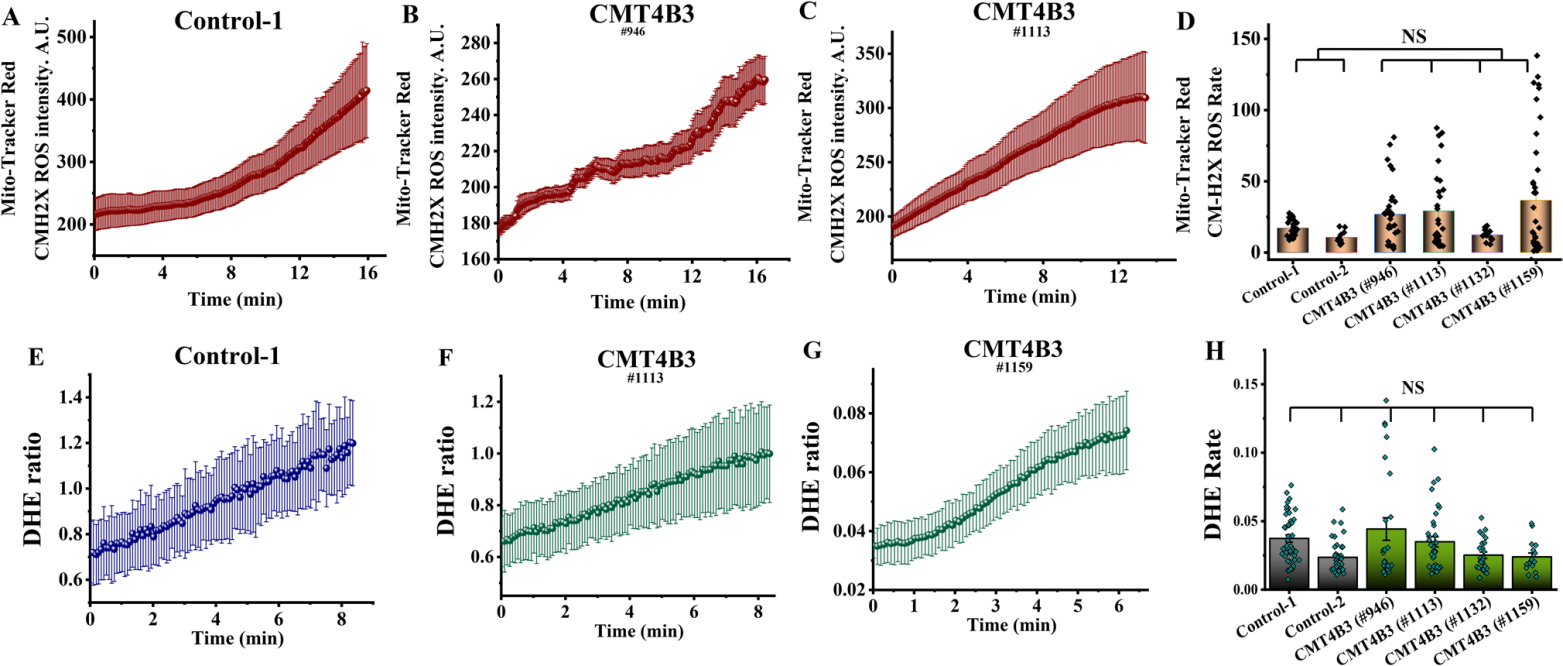
CMT4B3 pathology does not correlate with an excessive generation of reactive oxygen species (ROS). **A**–**D** Representative traces and histogram showing the rate of MitoTracker Red CM-H2XRos, indicating that the basal rate of mitochondrial ROS production patients’ cells is not significantly different from the rate in cells from controls. **E**–**G** Representative traces of the cytosolic ROS production as measured by the increase in DHE fluorescence. **H** Quantification of the rate of DHE fluorescence increase, indicating that basal rate of cytosolic ROS production is similar in cells of patients compared to controls. (n = 100; N = 4–6). Data are represented as mean ± SEM

Mitochondrial dysfunction may be associated with an increased ROS production in these organelles [11]. To identify the effect of CMT4B3-associated mutations on the rate of ROS production in mitochondria we used fluorescent indicator MitoTracker Red CM-H_2_XRos. In our experiments none of the patient’s fibroblasts with CMT4B3 pathology had any significant increase of the basal rate of mitochondrial ROS production compared to control (Fig. 4A–D). However, part of the mitochondrial ROS is produced outside of the matrix of mitochondria, and, many neurological conditions are also associated with an increase in ROS production in the cytosol [12, 13]. Using dihydroethidium (DHE) as an indicator for ROS production in the cytosol we have found that the rate of cytosolic ROS production in fibroblasts with CMT4B3-associated mutations was similar to the one measured in controls (Fig. 4E–H). Thus, CMT4B3 pathology is not associated with overproduction of ROS.

### CMT4B3 pathology led to massive decrease in the level of GSH

**Fig. 5:**
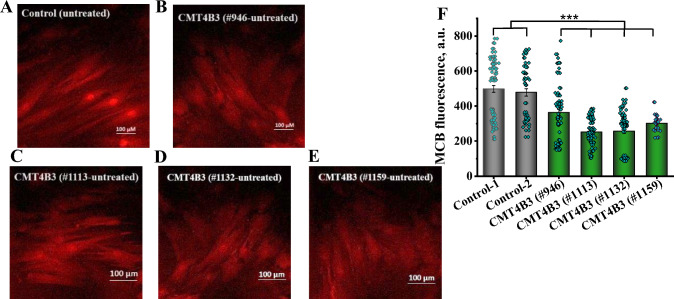
The level of glutathione is notably reduced in patient’ cells with CMT4B3 mutations. **A**–**E** Representative images showing the levels of glutathione in control and patient’ cell lines. MCB was used to visualize cytoplasmic GSH by live cell imaging. Scale bar: 100 μm. **F** Quantification of the level of MCB fluorescence, indicating that basal rate of cytosolic GSH production is significantly lower in cells from patients compared to controls (***p < 0.001). n = 100–200; N = 3–5

Glutathione (GSH) is the main endogenous antioxidant in the cells and the level of GSH is a direct indicator of the redox balance and oxidative stress. Using monochlorbimane (MCB) as a fluorescent indicator for intracellular GSH we have found that the level of GSH in the fibroblasts with CMT4B3 associated mutations is significantly lower than in control fibroblasts, in some lines reaching twofold decrease (Fig. 5A–F). Considering the low level of cytosolic and mitochondrial ROS production in fibroblasts with CMT4B3 pathology we can suggest possible alteration of the pathways for GSH synthesis.

### CMT4B3 mutations has no effect on glutathione reductase or glutathione synthase

**Fig. 6:**
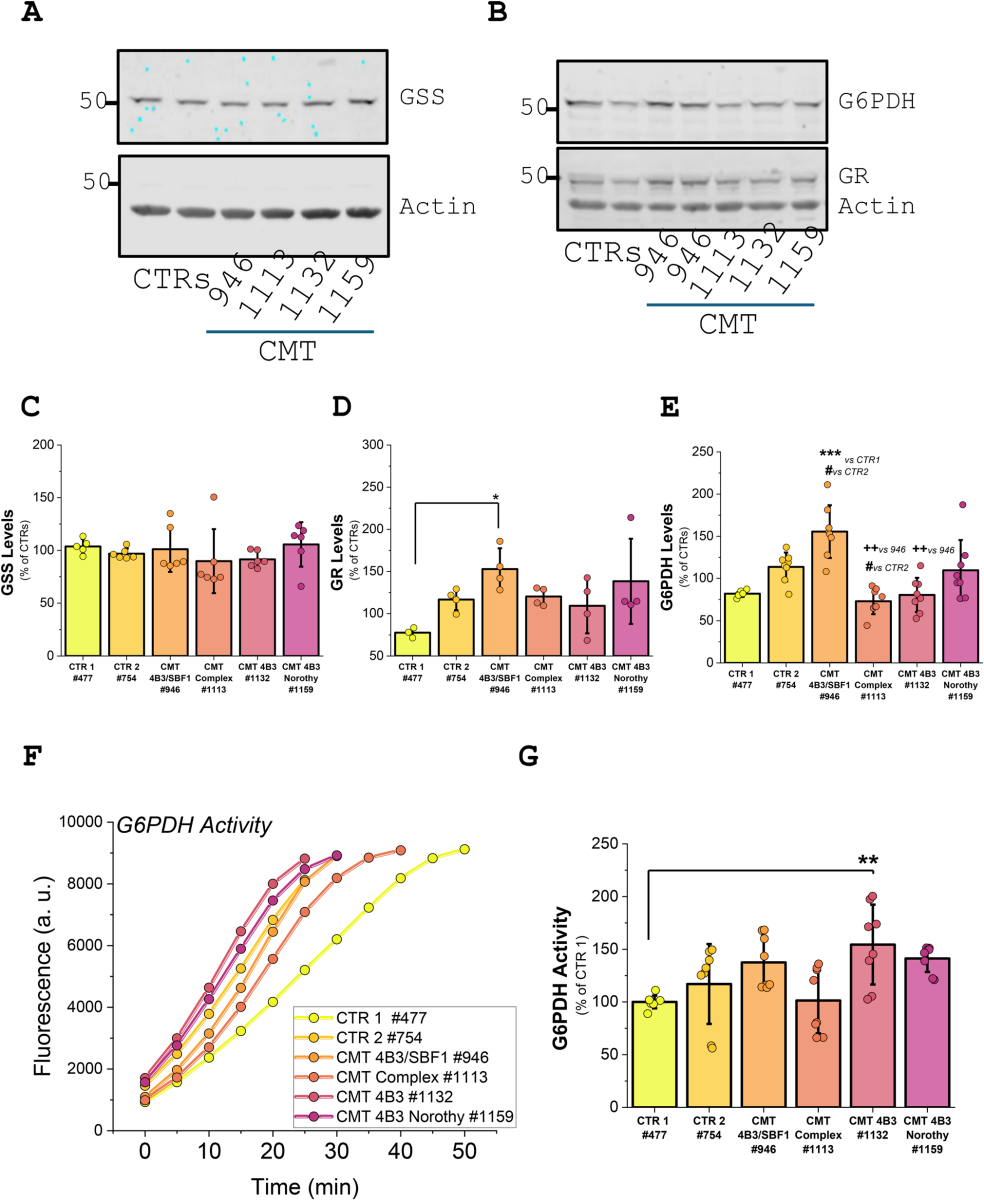
Glutathione homeostasis enzymes levels and activity are not altered in CMT4B3 pathology. **A**, **B** Representative blots showing glutathione synthetase (GSS), glutathione reductase (GR) and glucose-6-phosphate dehydrogenase (G6PDH) levels in controls and CMT fibroblasts. Actin was used a loading control. **C**–**E** Histograms show the relative (% of CTR) levels of GSS, GR and G6PDH normalized to actin (n = 4–8 individual samples in N = 5 independent experiments). **F** Representative traces and **G** quantification of G6PDH activity (as % of controls) in n = 8, N = 4 independent experiments. Data are reprented as mean ± SD. One-way ANOVA with Bonferroni post-hoc. *p < 0.05, **p < 0.01, *** *p* < 0.001

Considering the low level of glutathione in fibroblasts with pathology using western blot analysis we studied whether the level of major enzymes involved in the GSH maintenance–glutathione synthase (GSS) and glutathione reductase (GR) were changed in cells with CMT4B3 pathology. We have found that the expression level of GSS in fibroblasts with CMT4B3 mutations is almost identical to control (Fig. 6A, C). Interestingly, the level of GR was even higher in the patients’ fibroblasts with the SBF1 mutation (#946) while the rest of the patients’ cells were similar to control with trends to high GR (Fig. 6B, D). GR uses NADPH as a donor of electrons for the reduction of GSSG and the level of NAD(P)H in fibroblasts with CMT4B3 pathology in our experiments was also very low (Fig. 2F). Considering this, the low NADPH level could be the limiting factor for glutathione reduction that leads to the lower levels of GSH.

### CMT4B3 pathology changes the activity and expression levels of Glucose-6-Phosphate Dehydrogenase

Major producer of NAD(P)H is the pentose phosphate pathway (PPP) using the enzyme Glucose-6-Phosphate Dehydrogenase (G6PDH). In order to understand the significantly lower NAD(P)H and GSH level in the cells with CMT4B3 pathology we have measured the level of G6PDH in these cells as well as the activity of this enzyme. We have found that expression of G6PDH in patient’s fibroblasts varies in those cells (Fig. 6B, E). Production of NAD(P)H in G6PDH depends anyway on the activity of this enzyme. Activity of G6PDH in patients’fibroblasts was in general similar than in controls, except for one of the patients ((#1132) (Fig. 6F, G). The lower NAD(P)H (Fig. 2) and glutathione (Fig. 5) levels in these cells must then be explained by other mechanisms.

### Fibroblasts of patients with CMT4B3 pathology are vulnerable to exogenous ROS

**Fig. 7:**
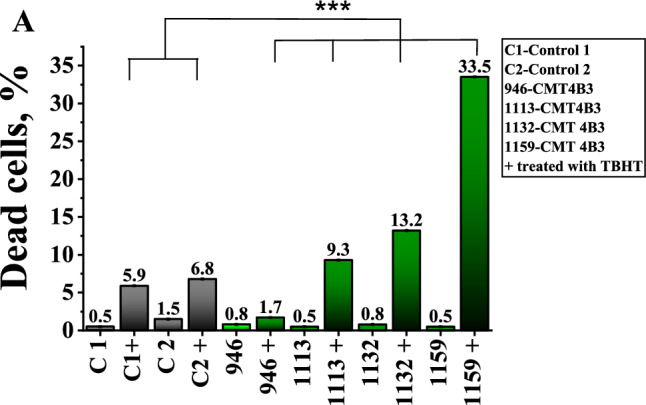
Vulnerability of the cells with CMT4B3 mutation to oxidative stress. **A** Histogram showing the percentage of cell death in controls and patients in the absence or presence (24 h) of the t-BHP, 50 μM. ***p < 0.001. Cell death was estimated by co-staining the cells with propidium iodide (red fluorescence) which labels dead cells, and Hoechst (blue fluorescence) which labels total number of cells. n = 800–1000; N = 3

Considering the mitochondrial dysfunction and oxidative stress due to a lower production of GSH, we studied the viability of the cells using PI as an indicator for dead cells. PI is impermeable for plasma membrane and binds with nuclear DNA and becomes fluorescent only if plasma membrane integrity is compromised. The total number of cells was identified with Hoechst 33,342. We have found that the percentage of dead cells among fibroblasts with CMT4B3 pathology is low and similar to the control (Fig. 7). We suggested that if CMT patients’ fibroblasts have a low endogenous antioxidant level, they should be very sensitive to a treatment with exogenous oxidants. To check this, we incubated these cells with t-BHP (500 μM) for 24 h. We have found that this treatment increased the percentage of the dead cells in both control lines of fibroblasts but much higher, up to 60 × in the fibroblasts from CMT4B3 patients, except in patient (#946), in which cell death was significantly lower than in the controls after the t-BHP treatment (N = 4 experiments; p < 0.001; Fig. 7).

## Discussion

Although CMT is a heterogeneous neurological disease mitochondrial dysfunction was shown to be associated with the most CMT-related mutations [20] including CMT4B3 [6]. In agreement with previously published data [6] we have also found CMT4B3-related mitochondrial abnormalities. Thus, in our experiments CMT4B3 decreases the mitochondrial membrane potential in the same way as it was shown for GDAP-1 [21, 22], Hsp27 [9] or CMT2L [23] mutations. However, a decrease in Δψm of previously published mutations was associated with the inhibition of complex I of ETC, inhibited mitochondrial respiration and an increase in mitochondrial ROS production. In fibroblast with CMT4B3 pathology we have found that decrease in Δψm is associated with the decreased mitochondrial NADH redox index and combination of these results strongly suggests mitochondrial uncoupling rather than inhibition of mitochondrial respiration. Lower rates of ROS production in mitochondrial also confirm this, because a mild uncoupling is widely used for attenuating higher mitochondrial ROS production [19]. Interestingly, lower ATP levels in CMT pathology was shown for various mutations [24, 25], in our experiments we have found that CMT4B3-induced mitochondrial dysfunction lead to shift cells to glycolysis. We can suggest that in the cells with higher energy demand such as neurons or myocytes CMT4B3 pathology can induce an ATP decrease and energy deprivation due to the lower rate of ATP production in glycolysis compared to the production in oxidative phosphorylation and consumption of ATP in complex V for Δψm maintenance. However, despite the mitochondrial dysfunction, disruption of the energy metabolism in the CMT4B3 fibroblasts was not the major trigger for pathology.

Maintenance of the redox homeostasis is important for redox signalling and protection of cells against oxidative stress [12]. Here we found that fibroblasts with CMT4B3 pathology have an overall lower rate of ROS production in mitochondria and cytosol. In contrast to these results, the level of GSH was significantly lower. Lower level of glutathione was shown previously for some other forms of CMT [26–28]. It should be noted that one of the CMT mutations—GDAP1 is an atypical glutathione S-transferase (GST) of the outer mitochondrial membrane, abnormal function of which could be a reason for the low GSH in those cells [28]. However, in our experiments the level of GSH, the two main enzymes responsible for GSH–glutathione synthase or glutathione reductase had a similar to control level.

The level of GSH is dependent on the NADPH concentration in the cells [29]. The level of NADPH in the cells with CMT4B3 pathology was significantly lower (Fig. 2). Major NADPH producer in the cells is pentose phosphate pathway (PPP) and specifically glucose-6-phosphate dehydrogenase (G6PDH) which catalyses the first and rate limiting step of the PPP, which has a crucial function in providing NADPH for antioxidative defence and reductive biosynthesis [30]. The expression level of G6PDH in the cells with CMT4B3 pathology was higher in one of the patients (946) compared to control, although the activity of this enzyme was unchanged in all, except in patient 1132. Since, glucose-6 phosphate is the rate limiting substrate for both pathways: glycolysis and PPP, thus when glycolysis is the major source of ATP in CMT4B3 fibroblasts there is a lack of substrate for both the NADPH production in PPP which in turn is crucial for GSSG reduction to GSH. Thus, the increased expression levels and activity of G6PDH may be one of the major attempt to compensate for decreased glutathione level in the CMT4B3 pathology.

Lower level of NADPH could be induced by limitation of glucose level due to higher demand of it in glycolysis and mitochondrial respiration [18]. However, this type of energy limitation could be trigger for cell collapse in the time of high energy demand processes–such a seizures or glutamate excitotoxicity [31, 32]

In agreement with our results, importance of GSH depletion in the mechanism of pathology of CMT shown in CMT4A, which is associated with mutation of the GDAP1 protein located on the outer membrane of mitochondria. Fibroblasts from CMT4A patients had low GDAP1 levels, decreased GHS concentration and a reduced mitochondrial membrane potential [15].

Combination of mitochondrial dysfunction and oxidative stress due to lower production of GSH may be one of the major reasons for CMT4B3 pathology and as we show here, that even relatively low level of ROS can induce cell death in cells with decreased endogenous antioxidants.
